# Nitric oxide treatments as adjuncts to reperfusion in acute myocardial infarction: a systematic review of experimental and clinical studies

**DOI:** 10.1007/s00395-016-0540-y

**Published:** 2016-02-24

**Authors:** Justin S. Bice, Bethan R. Jones, Georgia R. Chamberlain, Gary F. Baxter

**Affiliations:** Division of Physiology and Pharmacology, School of Pharmacy and Pharmaceutical Sciences, Cardiff University, Redwood Building, King Edward VII Avenue, Cardiff, CF10 3NB UK

**Keywords:** Nitric oxide, Ischaemia, Reperfusion, Systematic review, Myocardial infarction

## Abstract

Unmodified reperfusion therapy for acute myocardial infarction (AMI) is associated with irreversible myocardial injury beyond that sustained during ischemia. Studies in experimental models of ischemia/reperfusion and in humans undergoing reperfusion therapy for AMI have examined potential beneficial effects of nitric oxide (NO) supplemented at the time of reperfusion. Using a rigorous systematic search approach, we have identified and critically evaluated all the relevant experimental and clinical literature to assess whether exogenous NO given at reperfusion can limit infarct size. An inclusive search strategy was undertaken to identify all in vivo experimental animal and clinical human studies published in the period 1990–2014 where NO gas, nitrite, nitrate or NO donors were given to ameliorate reperfusion injury. Articles were screened at title and subsequently at abstract level, followed by objective full text analysis using a critical appraisal tool. In twenty-one animal studies, all NO treatments except nitroglycerin afforded protection against measures of reperfusion injury, including infarct size, creatinine kinase release, neutrophil accumulation and cardiac dysfunction. In three human AMI RCT’s, there was no consistent evidence of infarct limitation associated with NO treatment as an adjunct to reperfusion. Despite experimental evidence that most NO treatments can reduce infarct size when given as adjuncts to reperfusion, the value of these interventions in clinical AMI is unproven. Our study raises issues for the design of further clinical studies and emphasises the need for improved design of animal studies to reflect more accurately the comorbidities and other confounding factors seen in clinical AMI.

## Introduction

Early management of acute myocardial infarction (AMI) focuses on achieving rapid reperfusion of the ischemic risk zone in order to minimise irreversible tissue injury [65]. Although early reperfusion is undoubtedly beneficial after AMI, it can be associated with patterns of reperfusion injury. The deleterious effects of reperfusion on the myocardium occur as a result of the rapid reintroduction of oxygenated blood into the ischemic tissue. There are likely to be multiple underlying mechanisms of reperfusion injury but the most studied aspect is the formation of reactive oxygen species (ROS), in particular superoxide (O_2_^−^) and hydrogen peroxide [49]. These highly reactive species cause oxidative damage to the sarcoplasmic reticulum, mitochondria, cell membrane, nuclear DNA and sarcomeric proteins, leading to calcium overload of the cardiomyocytes [50] and opening of the mitochondrial permeability transition pore (mPTP) [14]. Ultimately, unmodified reperfusion is associated with cardiomyocyte apoptosis and accelerated necrosis of cells already damaged by ischemia. Furthermore, damage to the microvasculature causes a reduction in blood flow leading to the “no-reflow phenomenon” [55].

Nitric oxide (NO) is endogenously produced within myocardium, principally from l-arginine under the influence of nitric oxide synthases (NOS). It can also be produced via NOS-independent mechanisms including the reduction of tissue reservoirs of nitrite (NO_2_^−^) or nitrate (NO_3_^−^) to liberate NO under hypoxic conditions [6], such as occurs in the ischemic myocardium. The production of NO from NO_2_^−^ has been shown to reduce myocardial injury [8, 33] and the reduction of NO_2_^−^ is thought to be facilitated by molecules including deoxymyoglobin [5] and the enzyme xanthine oxidoreductase [66] among others. NO has a short half-life in vivo and the conversion of NOS derived NO into a variety of storage forms by oxidase enzymes [57] is an important reservoir of NO.

Nitric oxide has been shown in many experimental studies to modulate ischemia/reperfusion injury. Administration of NOS inhibitors has been reported to exacerbate myocardial necrosis [23] supporting the notion that endogenous NO is protective against ischemia/reperfusion injury [18]. In experimental studies, endogenous NO has been shown to contribute in the protective pathways activated in classical and delayed ischemic preconditioning [10] and also hibernation [19]. These potential protective effects of endogenous NO have given rise to a multitude of experimental and clinical studies focusing on the delivery of exogenous NO, in the form of various NO species and NO-donor compounds, to limit ischemia/reperfusion injury [7] with the general hypothesis being that NO ameliorates ischemia/reperfusion injury.

The current study addresses the question of whether NO treatments/namely gaseous NO, NO_2_^−^, NO_3_^−^ or organic NO donor compounds, as adjuncts to reperfusion following ischemia, provide consistent cardioprotection against reperfusion injury, when assessed primarily as a reduction in infarct size. We addressed this question by undertaking a systematic qualitative review of experimental and clinical studies that have investigated the effects of NO treatments, when given specifically in a manner that could modify reperfusion injury in (a) in vivo animal models of ischemia/reperfusion or (b) in patients undergoing reperfusion therapy for AMI. We identified articles against predefined, highly selective inclusion criteria and critically analysed relevant articles to evaluate the quality of the studies. Those studies subjected to full text analysis were then synthesised to form the basis of this review.

## Methods

### Study design

The study design was based on the preferred reporting items for systematic reviews and meta-analyses (PRISMA) 27-point guidance [36] together with review protocols published by the Cochrane Collaboration [1]. A systematic methodological approach was designed in order to reduce reviewer bias when selecting articles for inclusion and to appraise the included articles against predefined inclusion criteria to create an objective synthesis of the current published data.

### Search strategy

Following a pilot study to scope the approximate period and scale of the relevant scientific literature, search terms were agreed by all reviewers (BRJ, GRC, JSB, GFB). Terms were expanded with Boolean operators, as well as adjacency and tree techniques, in order to combine search terms and narrow the specific literature to be included. Due to differences in search functionality between the databases some search terms were adapted or omitted for individual databases. Two reviewers (BRJ, GRC) used the final agreed search terms to search the Cochrane library, Medline, Embase, Web of Science, and the clinical trials databases CT.gov. The strategy was limited exclusively to exogenous sources of NO and their specific effects on myocardial reperfusion injury; as such, terms relating to precursors such as l-arginine and endogenous NO, or pre-ischemic treatment were excluded.

### Eligibility criteria

We included all animal and human studies utilising exogenous administration of gaseous NO, organic NO-donors, NO_2_^−^, NO_3_^−^ or OONO^−^ during periods relevant to reperfusion injury. Original articles in the serial literature published in English during the period January 1st 1985 to August 15th 2014 were included. Review articles were screened to identify relevant publications once the articles reached the full text level of the screening process. We did not search university dissertation or thesis repositories. Published outputs were included if they met the criteria listed in Table 1.

**Table 1:** Inclusion criteria

**Criteria for inclusion of published animal studies**	
(a) Peer reviewed original article (b) In vivo animal study (c) Conducted on suitable animal species with characterised levels of collateralisation of the coronary circulation (rodents, rabbits, pig, cats, and dogs) (d) Documented period of ischemia (e) Documented period of reperfusion (f) Intervention group in which animals were administered a documented NO treatment (regardless of route of administration) within the latter stages of the ischaemic phase or in the early reperfusion phase (g) Clearly defined contemporary control group where animals received defined control treatment (h) Infarct size measured as endpoint by clearly documented method	
**Criteria for inclusion of published human studies**	
(a) Peer reviewed original article (b) Documented period of myocardial ischemia (time from onset of chest pain) (c) Documented method of reperfusion (d) Intervention group in which patients were administered documented NO treatment (regardless of route of administration) prior to, or during PCI/thrombolysis (e) Completed randomised control trial with infarct size estimation as clearly defined endpoint	

A critical appraisal tool was developed to allow a comprehensive qualitative critique of the articles at full text level (Table 2).

**Table 2:** Critical appraisal tool

(a) Details about study population including numbers in each treatment group and baseline characteristics (b) Details regarding intervention and control arms of the study (c) Specific endpoints being reported and how they were assessed (d) Whether randomisation of study participants took place (e) Timing of administration of the intervention being investigated (f) Reporting of study protocols such as methods and timings of ischemia and reperfusion (g) Assessment of sample size and power of study (h) Whether inclusion/exclusion criteria for study or its participants were stated (i) Whether methods of data analysis used were appropriate for data types being reported (j) Whether reporting of results was accurate and conclusion of study reflected results reported (k) Whether limitations of study or conflicts of interest were acknowledged by authors	

### Article selection and data extraction

Article screening was undertaken in a standardised, non-blinded manner by the two primary reviewers (BRJ, GRC) who independently screened the search results for relevance by reviewing the titles (11,539) and subsequently the abstracts (548) of the identified studies using the eligibility criteria. Following relevance screening, the two reviewers compared results to identify any disagreements or queries and the secondary reviewers (JSB, GFB) gave input until a consensus was reached. All articles deemed relevant (58 animal studies and 35 human studies) underwent full text critical analysis independently by BRJ and GRC who concluded which articles were appropriate for inclusion (Table 3). Each of the articles was then discussed between the primary reviewers and any disagreements were resolved by the secondary reviewers (JSB, GFB). The finalised included and excluded articles were then sampled by the secondary reviewers (JSB, GFB) to confirm the consistency of the data analysis process. Once the sampling process had been completed, the included studies (21 animal and three human) were critiqued.

**Table 3:** Exclusion of articles

Reasoning	No. articles
Excluded during relevance screening (title plus abstract) level	11,539
Total no. of articles appraised at full text level	93
Excluded during full manuscript review	
Inappropriate timing of NO donor administration	24
Inadequate/lack of suitable control arm	1
No clear period of ischaemia and/or reperfusion stated	12
NO donation not primary mechanism of action being investigated	8
Ex vivo/in vitro study	4
Inappropriate outcomes measured	11
Not myocardial I/R injury	3
Abstract or preliminary results	4
Review article	1
Foreign language article	1
No. of studies excluded at full text level	69
No. of studies included after full text evaluation	24

### Meta-analysis

A random effects model was used as it was considered that heterogeneity would be demonstrated due to varying treatments and animal models. Data are reported as mean difference. Authors were contacted to clarify data values if SEM or SD were not published. Statistical heterogeneity was determined using *I*^2^. Sub analysis of grouped studies [by species, NO donor (data not shown)] did not cause significant deviation from the mean difference reported here.

## Results

### Study inclusion/exclusion

**Fig. 1:**
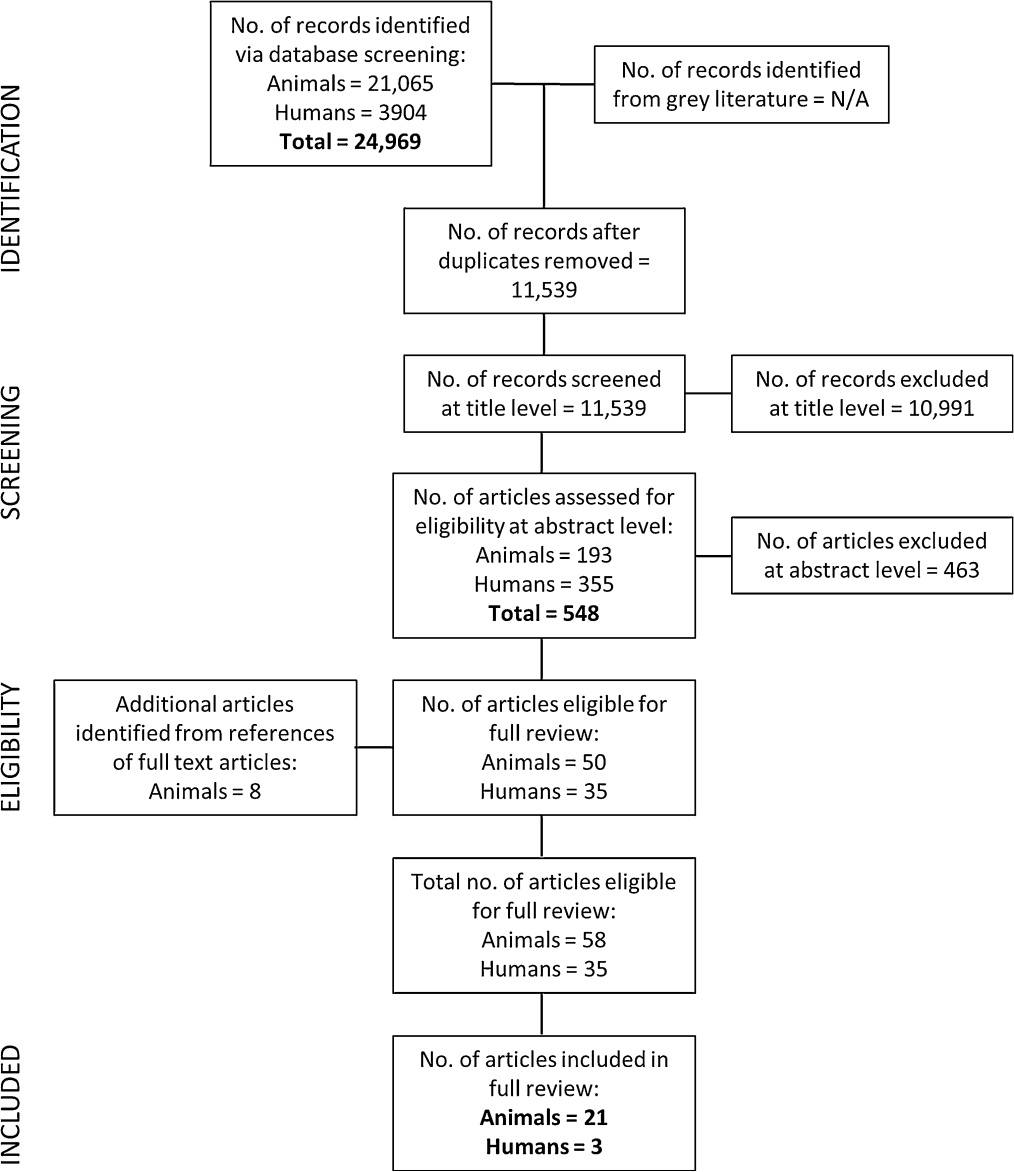
Results of database searches and appraisal at different stages of the review process

The results of the article selection and data extraction process are summarised in Fig. 1. The database search provided a total of 24,969 citations (from both animal and human studies), and after removing duplicate reports, 11,539 remained of which 10,991 were discarded at title level, leaving 548 articles. Of these, 463 studies were excluded at abstract level since they did not meet the eligibility criteria (Table 1). The remaining 50 animal studies and 35 clinical studies were obtained in full text and an additional eight animal studies were identified from the reference lists (“snowballing”) of the remaining animal studies. A total of 58 animal and 35 human studies were therefore appraised at full text level (see Fig. 1). Following this appraisal, 37 animal and 32 human studies were not deemed to have appropriate methodology or quality for inclusion, leaving 21 animal and three human studies to be included in the two arms of the review (Table 3).

### Characteristics of experimental animal studies

Table 4 summarises the characteristics and outcomes of 21 in vivo animal studies analysed. Animal studies examined the role of NO treatments in anesthetised animals subjected to ischemia/reperfusion with infarct size assessment as a major endpoint. The species used were cat, dog, mouse, rat, rabbit and pig. In the majority of studies, myocardial ischemia was induced by reversible ligation of the left anterior descending coronary artery (or similar in rodents). A few studies used alternative methods for induction of ischemia including clamping of the aorta or inflation of a balloon in the coronary artery. Duration of ischemia ranged from 30 to 120 min with reperfusion lasting between 2 and 24 h. Injury was assessed by infarct size determination, predominantly by TTC contrast staining.

**Table 4:** Summary of included experimental studies

Author, year	Animal species	Exp. protocol/primary endpoint determination	NO donor	Timing of NO administration	*n* (Tx):*n* (control)	Effect of NO donor on outcome vs control
Lefer et al. (1993)	Adult male cats	LAD occlusion 90 min Reperfusion 270 min Endpoint: IS − TTC	Tx: novel sydnonimine NO donor C87-3754 (1 mg/kg/h) Control: non-NO donating analogue C88-3934 IV infusion	10 min before reperfusion until end of experiment	6:6	↓ % IS/AAR compared to control (12 vs 33 %)
Hataishi et al. (2006)	2–4 month old wild type mice	LCA occlusion (a) 30 min, (b) 60 min, (c) 120 min Reperfusion 24 h Endpoint: IS − TTC	Tx: iNO 80 ppm Control: inhalation of O_2_ (0 ppm N_2_) Mechanical ventilator	20 min before reperfusion until end of experiment	(a) 13:14 (b) 6:7 (c) 9:11	iNO 80 ppm ↓ IS/AAR % after 30, 60 and 120 min ischaemia iNO dose–response 40/80 ppm = ↓IS/AAR % but not 20 ppm iNO
Duranksi et al. (2005)	Mice 8–10 week old	LCA occlusion 30 min Reperfusion 24 h Endpoint: IS − TTC	Tx: (a) NaNO_2_ 48 nmol (b) NaNO_2_: 2.4, 4.8, 960 and 1,920 nmol Control: 48 nmol NaNO_3_ Intraventricular	Admin into LV cavity 5 min prior to reperfusion	*a* = 8 *b* = 6 per dose Control = 11	All NO_2_^−^ doses (except 1,920 nmol) = significant ↓ IS/AAR % compared with control 48 nmol nitrite significantly (*P* < 0.001) reduced IS compared to control
Hendgen-Cotta et al. (2008)	Mice 14 ± 3 week old	LCA occlusion 30 min Reperfusion 24 h Endpoint: IS − TTC	Tx: NaNO_2_ 48 nmol Control: 48 nmol NaNO_3_ Intraventricular	Admin into LV cavity 5 min prior to reperfusion	7:7	48 nmol nitrite significantly (*P* < 0.01) reduced IS compared to control
Johnson et al. (1990a)	Adult male cats	LAD occlusion 1.5 h Reperfusion 4.5 h Endpoint: IS − TTC	Tx = acidified NaNO_2_ in 0.12 M HCl at pH 2.0 (a) 50 mmol/kg/h (b) 25 mmol/kg/h (c) 12.5 mmol/kg/h Control: acid vehicle IV infusion	30 min after induction of ischaemia until end of reperfusion	Sham:7 Control = 6 *a* = 7 *b* = 6 *c* = 6	*a* = maximal cardioprotection Inf. rates of <12.5 mmol/kg/h provide NS protection IS/AAR significantly ↓ in NaNO_2_ (*a* + *b* + *c*) compared to vehicle treated groups
Baker et al. (2007)	Male rats 8 weeks old	LCA occlusion 30 min Reperfusion 2 h Endpoint: IS − TTC	Tx: NaNO_2_ 4 mg/kg IV at time of admin until end of reperfusion phase Control: saline IV infusion	(a) NO_2_^−^ 15 min after ischaemia (b) NO_2_^−^ 10 secs after reperfusion	6:6:6	NaNO_2_ admin in (*a*) produced significant ↓ IS/AAR compared to control NaNO_2_ admin in (*b*) shows no significant ↓ in IS
Johnson et al. (1990)	Adult male cats	LAD occlusion 1.5 h Reperfusion 4.5 h Endpoint: IS − TTC, serum CK levels	Tx: acidified NaNO_2_ (pH 2.0), 12.5 mmol/kg/h Control: acid vehicle IV infusion	30 min post-occlusion until end of reperfusion	6:6	Significant ↓ IS in Tx group compared to control
Lefer et al. (1993b)	Dogs (M/F)	LAD occlusion 60 min Reperfusion 270 min Endpoint: IS − TTC	Tx: novel cysteine containing mononitrate NO donor (SPM-5185) Control: NO deficient analogue (SPM-5267) IV infusion	After 60 min of ischaemia, IV infusion to achieve plasma conc of 500 nM	6:5	Highly significant ↓ IS/AAR % in Tx group (14.5 %) compared to control (47.5 %)
Tripathi et al. (1997)	Adult male mongrel dogs	LAD occlusion 90 min Reperfusion 4 h Endpoint: IS − TTC, VF − ECG	Control: saline reperfused Tx: acidified NaNO_2_ infusion 0.30 Mol/L HCl pH 2 IV infusion	Saline or NaNO_2_ infused at time of reperfusion for 4 h	10:10	NS diff in NaNO_2_ vs saline Tx groups in % IS/AAR or LV
Liu et al. (2007)	Juvenile pigs (M/F)	Balloon-mounted stent for 50 min Reperfusion 240 min Endpoint: IS − TTC	Tx: iNO: 80 ppm Tx: IV-NTG: 2 µg/kg/min Control: IV saline IV infusion	10 min before reperfusion until end of experiment	Saline = 14 iNO = 12 IV-NTG = 11	IV-NTG did not significantly ↓ IS/AAR compared to control iNO ↓ IS by 47 % compared to control
Lefer et al. (1993a)	Canines (M/F)	LAD occlusion 60 min Reperfusion 270 min Endpoint: IS − TTC	Tx: novel cysteine containing mononitrate NO donor (SPM-5185) Control: saline IV infusion	60 min of ischaemia throughout reperfusion	10:7	↓ IS in Tx group (SPM5185 = 3.1 %, control = 13.6 %)
Nossuli et al. (1997)	Adult male cats	LAD occlusion 90 min Reperfusion 4.5 h Endpoint: IS − TTC	ONOO^−^ 1 µmol/L in pH 8.4 saline as Tx group Control = pH 8.4 saline Intra-ventricular or IV infusion	10 min prior to reperfusion until end of experiment	6:6	Significant ↓ in IS/AAR (*P* < 0.001) and necrosis/LV (*P* < 0.02) in Tx group compared to control
Shinbo et al. (2013)	10 week old male mice	LCA occlusion 60 min Reperfusion 24 h Endpoint: IS − TTC	Tx: iNO 80 ppm gas Control:FiO_2_ 0.3 Inhaled via mechanical ventilator	5 min prior to reperfusion until end of experiment	5:5	IS/AAR significantly ↓ in iNO mice compared to control
Nagasaka et al. (2008)	Male mice	LCA occlusion 60 min Reperfusion 24 h Endpoint: IS − TTC	Tx; iNO 80 ppm Control: mice breathing O_2_ Inhaled via mechanical ventilator	iNO administered during ischaemia for (before reperfusion): (a) 60 min (b) 5 min (c) 0.5 min	(a) 9:10 (b) 8:9 (c) 6:7	*a* = ↓ IS/AAR by 32 % compared to O_2_ mice (*P* < 0.05) *b* = ↓ IS/AAR by 31 % compared to O_2_ mice (*P* < 0.05)
Nagasaka et al. (2011)	WT mice 8-12 week old Mice with sGC_α1_^−/−^ deficiency	LCA occlusion 60 min Reperfusion 24 h Endpoint: IS − TTC	Tx: iNO 80 ppm Control: O_2_ inhalation Inhaled via mechanical ventilator	60 min beginning 10 min after LCA occlusion until 10 min reperfusion	WT = 10:12 sGC_α1_^−/−^ = 10:10	iNO Tx in WT mice caused 41 % ↓ in MI/AAR (*P* < 0.001), however did not alter MI/AAR in sGC_α1_^−/−^ mice
Pabla et al. (1995)	Mongrel dogs (M/F)	LAD occlusion 90 min Reperfusion 270 min Endpoint: IS − TTC	Tx: long acting NO donor: CAS-1609 IV bolus 1.25 mg, followed by infusion of 1 mg/h Control: normal saline bolus and infusion IV infusion	Bolus 10 min before reperfusion followed by infusion for reperfusion period	7:7	IS/AAR in Tx group = 8 %, control = 29 % (*P* < 0.01) (70 % ↓ in necrosis)
Salloum et al. (2007)	Male NZ white rabbits	LCA occlusion 30 min Reperfusion 3 h Endpoint: IS − TTC	Tx: NTG 2 µg/kg/min IV via continuous infusion Control: 0.9 % saline IV infusion	5 min prior to reperfusion continuing for 65 min	7:6	NS diff in IS/AAR between NTG and saline groups (31.5 vs 33.8 % respectively)
Nossuli et al. (1998)	Adult male cats	LAD occlusion 90 min Reperfusion 270 min Endpoint: IS − TTC	Tx : ONOO^−^ infusion in pH8.4 saline at: (a) 0.2 µM (b) 2 µM (c) 20 µM Control = pH 8.4 saline Route: IV infusion	Intraventricular infusion admin 10 nmin prior to reperfusion and maintained throughout reperfusion	Control = 6 *a* = 6 *b* = 7 *c* = 6	Only 2 µM dose of ONOO^−^ significantly ↓ IS/AAR %, (14.4 % in Tx compared to 30.3 % control *P* < 0.01)
Neye et al. (2012)	Male rats	LCA occlusion 120 min Reperfusion 3 h Endpoint: IS − TTC	Tx: iNO 50 ppm Control: room air Inhaled via mechanical ventilator	(a) iNO/control admin throughout 3 h reperfusion (b) iNO/control admin throughout 5 h period of I and R	8:8	*a* = IS/LV ↓ in iNO compared to control however IS/AAR was NS *b* = IS/LV was significantly ↓ compared to control and group a
Siegfried et al. (1992)	Adult male mongrel cats	LAD occlusion 90 min Reperfusion 270 min Endpoint: IS − TTC	*a* = NO donor: SIN-1 *b* = NO donor:C87-3754 *c* = inactive NO donor: C88-3934 *d* = control: 0.9 % saline Bolus 1 mg/kg followed by 1 mg/kg/h infusion IV	Administered into jugular vein 10 min prior to reperfusion until end of reperfusion period	*a* = 8 *b* = 6 *c* = 6 *d* = 6	*a* = ↓ IS/AAR from 29 to 9 % (*P* < 0.001) *b* = ↓ IS/AAR from 31 to 11 % (*P* < 0.001)
Johnson et al. (1991)	Adult male cats	LAD occlusion 90 min Reperfusion 270 min Endpoints: IS − TTC	*a* = NO in solution *b* = vehicle 1.1 mL/kg/h IV	30 min after LAD ligation until end of reperfusion period	*a* = 6 *b* = 6	*a* = ↓ IS/AAR from 26 to 7 % (*P* < 0.01)

*LAD* left anterior descending coronary artery, *LV* left ventricle, *AAR* area at risk, *MI* myocardial infarction, *PPM* parts per million, *IS* infarct size, *CK* creatinine kinase, *NTG* nitroglycerin, *HR* heart rate, *BP* blood pressure, *NaNO*_*2*_ sodium nitrite, *NaNO*_*3*_ sodium nitrate, *ONOO* ^*−*^ peroxynitrite, *PRI* pressure rate index, *TTC* triphenyltetrazolium chloride, *NO*_*2*_^*−*^ nitrite, *NO* nitric oxide, *HCl* hydrochloric acid, *SNP* sodium nitroprusside, *LCA* left coronary artery, *ROS* reactive oxygen species, *iNO* inhaled nitric oxide, *LVSP* left ventricular systolic pressure, *NS* not significant, *cGMP* 3′, 5′-cyclic guanosine monophosphate, *VF* ventricular fibrillation

Six studies administered inhaled NO (iNO), six administered sodium nitrite (NaNO_2_), five administered novel organic NO donors, two administered ONOO^−^, one administered sodium nitroprusside (SNP) and one administered nitroglycerin (NTG). Routes of administration of these agents included inhaled, intravenous and intraventricular administration, with timings of administration ranging from time points during ischemia but before reperfusion, to 10 s after the point of reperfusion. The concentration of NO treatments varied according to which agent was used (e.g. iNO 20–80 ppm, NaNO_2_ 2.4 nmol–12.5 mmol kg/h, ONOO^−^ 0.2–20 µM).

**Fig. 2:**
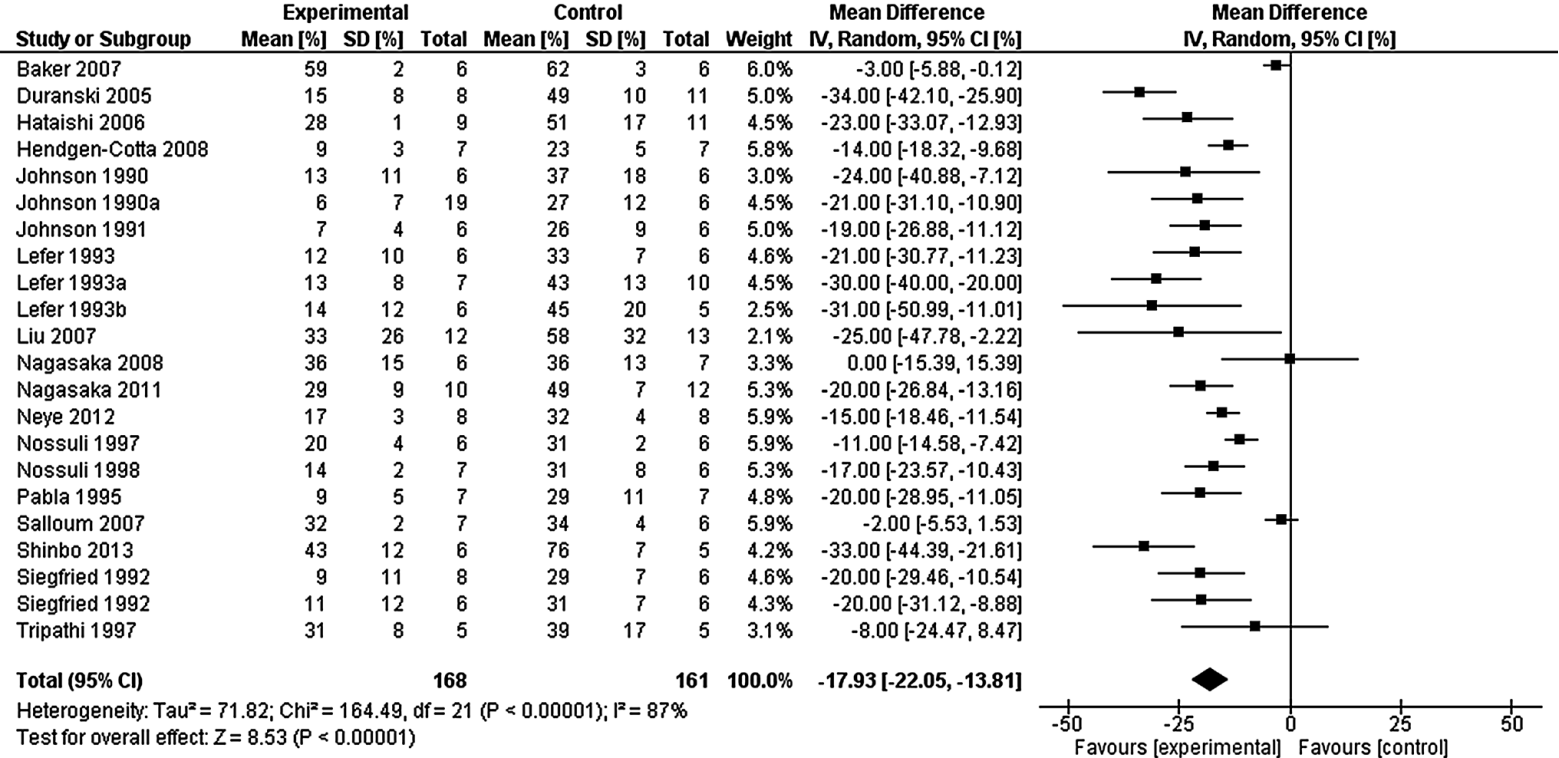
Infarct size in animal models in groups treated with NOx adjuncts compared to control experiments. N. B. NTG treatment for Liu et al. [37] not reported as no separate control group

Meta-analysis of all experimental studies suggests that infarct size was limited compared to relative controls [mean difference of −17.93 % (95 % confidence interval: −22.05, −13.81)] (Fig. 2) except when NTG was administered. Sensitivity analysis demonstrated that grouping of publications to animal model or specific NOx had little effect on the outcome of the analysis (data not shown). Statistical heterogeneity was high in all sub-group analysis, yet the mean difference in effect size was consistently similar.

### Characteristics of human clinical studies

The characteristics and outcomes of the three clinical studies which met the criteria for analysis are summarised in Table 5. The earlier studies by Hildebrandt et al. [21] and Morris et al. [37] administered isosorbide dinitrate over 24–48 h whilst in the most recent NIAMI study [59] NaNO_2_ was administered as a bolus. Reperfusion therapy in the earlier studies was carried out by thrombolysis in contrast to the NIAMI trial in which patients received PPCI 5 min after sodium nitrite. There was no reduction in infarct size in human studies following NOx administration.

**Table 5:** Summary of included clinical studies

Author, year	NO donor (dose, route, duration)	Time from onset of chest pain to admission (h)	Reperfusion technique	Infarct size determination	*n* (Tx):*n* (control)	Effect of NO donor on outcome vs control
Hildebrandt et al. (1992)	Isosorbide dinitrate 1.0–10.0 mg/mL Infusion for 48 h	≤8	Thrombolysis with streptokinase	CK-MB every 4 h for 72 h	50:49	No reduction in infarct size when reperfusion confirmed
Morris et al. (1995)	Isosorbide dinitrate 1.0–6.0 mg/h Infusion for 24 h minimum	≤24	Thrombolysis	αHBDH blood samples every 12 h on days 1 and 2 and daily on days 3, 4, and 5	150:151	No reduction in infarct size, ventricular remodelling or ST segment resolution at day 3
Siddiqi et al. (2014)	Sodium nitrite 70 μmol Infusion for 5 min	≤12	PPCI	CMR % LV mass 6–8 days post infarct	118:111	No reduction in infarct size or secondary endpoints including ejection fraction and troponin 1

PPCI primary percutaneous coronary intervention CMR cardiac magnetic ressonance HBDH hydroxybutyrate dehydrogenase CK-MB creatine kinase-MB

## Discussion

### Experimental animal studies

The key finding of the 21 in vivo animal studies critically reviewed is that, with the exception of NTG, NO treatment prior to or during the early reperfusion period can limit infarct size. However, considerable heterogeneity of effect was observed, related to both treatment (agent, dose, regimen) and species (notably whether collateralised or not).

Our analysis of the combined effects of all animal studies used a random-effects model and was reported as mean difference. Although random-effects models typically provide larger confidence intervals, the assumption made here was that studies were heterogeneous but effects followed some distribution. Indeed the analytical approach here provides an answer to the question “what is the average intervention effect?” The large degree of statistical heterogeneity is likely due to the differences in animal model and NO treatment utilised. However for the purposes of this review, in which we are interested in the overall picture, a summary effect of all interventions provides meaningful insight into targeting NO signalling in I/R.

### Sydnonimine nitric oxide donors

Two sydnonimine NO donors, C87-3754 and SIN-1, produced a marked reduction in infarct size compared to both conventional controls and non-NO donating analogues [29, 60] suggesting that protection is afforded by NO, when administered at relatively low doses (1 mg/kg/h IV). However both studies were conducted in cats, a species with a collateralised coronary circulation [34]. Collateralisation does not completely prevent infarction, but may alter processes during early ischemia [15] so modifying infarct size. Conversion of sydnonimines to release NO is sensitive to low pH, conditions found during early reperfusion [54]. Their use in contemporary studies is limited and haemodynamic profile in I/R unreported, however treatment exhibits a reduction in endothelial dysfunction, likely caused by NO quenching of free radical species [60].

### Inhaled gaseous NO

iNO significantly reduced infarct size at concentrations ranging from 40 to 80 ppm [16, 32, 38–40, 56] as well as decreasing creatine kinase (CK) concentrations and rate of apoptosis of cardiomyocytes [32] which was seen even when iNO was administered during short periods (e.g. 5 min prior to reperfusion) [39]. However beneficial effects were not seen when iNO was administered immediately before reperfusion. Therefore it is possible bioactive carriers of NO, such as nitrite [12] and S-nitrosylated [62] proteins, provide protective effects rather than molecular NO itself. Indeed the mechanism by which iNO is converted to a more stable nitrogen oxide molecule before entering the blood stream and eliciting extra-pulmonary effects remains to be fully elucidated [41]. The suitability of inhaled NO as an adjunct to reperfusion in the clinic is therefore questionable.

### Nitrite

NO_2_^−^ was shown to exert a dose dependent infarct-limiting effect, which peaked at 48 nmol when administered intraventricularly, providing significant reduction in infarct size compared to control [8]. However, the control treatment used in this study was NO_3_^−^, which was previously shown to exert a beneficial effect at high doses [27]. A contemporary study by the same group using similar timings of reperfusion showed comparable infarct size for a vehicle control group, suggesting that NO_3_^−^ at a concentration of 48 nmol had no cardioprotective effect over control. These results are corroborated by a more recent study by Hendgen-Cotta et al. who further demonstrated that 48 nmol NaNO_2_^−^ could limit infarct size in mice [17].

When NO_2_^−^ was co-administered with an NO scavenger, cardioprotection was abolished, suggesting the beneficial effects are NOS independent but NO-dependent [8]. However, despite studies showing NO_2_^−^ to be beneficial, when administered at the point of reperfusion it exerted no significant effect on infarct size when administered immediately after reperfusion [2] yet LV function after AMI was preserved [64]. This may be due to a difference in timing of administration, or possibly due to differences between rodents and dogs; the latter have a variably collateralised coronary circulation. Another possible interpretation may be the time for the nitrite species to be converted into a cytoprotective nitrogen oxide species if the mechanism of cyoprotection is not mediated by s-nitrosylation (for a comprehensive review of nitrite mediated protection the reader is directed to Rassaf et al. [52]). Acidified NaNO_2_ and NO in solution have also been demonstrated to limit infarct size in feline models of LAD occlusion [25, 26].

### Peroxynitrite

ONOO^−^ is formed when NO reacts with O_2_^−^ [35] and shows protective effects when administered at low micromolar concentrations while increasing infarct size at higher concentrations [44]. Maximal physiological concentrations have been previously documented in the order of 2–5 µM [44, 45]. A significant reduction in infarct size was observed when ONOO^−^ was administered via intraventricular infusion. However when infused intravenously no cardioprotection was afforded [45], suggesting ONOO^−^ acts locally rather than systemically. Furthermore, the short half-life and immediate interaction with plasma proteins such as glutathione would suggest that intravenous injection would fail to elicit the same response. Production of S-nitrosothiols from ONOO^−^ to from more stable nitrogen oxide resevoirs is a possible mechanism for affording cytoprotection [43]. The generation of ONOO^−^ during early reperfusion from ROS and NO and further ROS induced ROS release suggest that ONOO^−^ may not be suitable as a therapeutic agent.

### Other nitric oxide donor compounds

Several studies have suggested that novel NO donors may have advantages, such as increased potency and reduced tolerance compared to traditional NO donors [3, 31]. However whether this is of relevance to the setting of ischemia/reperfusion is unclear, as generally agents are not administered over long periods of time. Nevertheless all studies using other donors showed a significant reduction in infarct size [29–31, 47, 60]. There were however discrepancies in the results with respect to neutrophil accumulation and activation: this was seen in all the other NO donor studies, except the work by Siegfried et al. [60], and the animal model used (feline or canine) is a potentially confounding factor. Lefer et al. [29, 30] diverted coronary collateral blood flow away from the ischaemic area by inserting an open cannula through the arteriotomy distal to the occluded LAD and therefore suggested that the protective effect occurred independently of collateral blood flow. However other studies that utilised feline myocardial models failed to measure collateral flow and so it is difficult to conclude whether this would have contributed to infarct limitation at reperfusion. It may therefore be more appropriate to consider these agents with respect to a more representative animal model, such as pig in the future.

### Traditional nitric oxide donating compounds

In two studies, NTG did not reduce infarct size when administered at reperfusion [32, 53] which may be due to tolerance induced through continuous infusion or due to a relative reduction in its bioavailability [32]. There is sustained contradiction as to precisely how NTG causes vasodilation via NO signalling i.e. cGMP or nitrosylation. At clinical plasma concentrations evidence suggests that free NO is not released [46], but possibly a mechanism by which NTG nitrosylates other proteins which may lead to its vasoactive actions, a similar mechanism to that proposed for NTG tolerance following chronic administration [61]. Interestingly, NTG could afford late preconditioning in conscious rabbits, an observation that was sustained in NO tolerant rabbits [22].

### Downstream targets

These data support the overriding thesis that NOx is a successful candidate for targeting the injurious effects of ischaemia reperfusion injury in animal models. Evidence that suggests that endogenous production and maintenance of cofactors of NOS are compromised during injury, and the consequential reduction in NO bioavailability further supports this rationale. Addition of both l-arganine and tetrahydrobiopterin just prior to reperfusion in both rats and swine limit infarct size [63]. Increased NO availability and the subsequent reduction in superoxide production provides favourable conditions. Arginase inhibition has similarly been shown to limit infarct size by increased NO production [13].

Modification of the electron transport chain by S-nitrosation has also been well documented as a means of cytoprotection, ultimately inhibiting mitochondrial transition pore opening and reducing cyctochrome-c release [17, 58]. The reduction in pH and hypoxic environment during ischaemia favours nitrite reduction providing an environment suitable for NO_2_^−^ to afford infarct limitation by targeting complex I. Furthermore, NO has been shown to regulate the respiratory complexes and improve myocardial oxygen consumption [4]. Cyclophilin D can be S-nitrosylated at Cys^203^ which results in a reduction in mPTP opening in mouse fibroblasts, which is critical in reducing cell death [42].

### Human clinical trials

Three high quality clinical studies which met the criteria for inclusion were identified. The primary endpoint in all three studies was infarct size; there was no evidence of infarct size reduction in patients treated with NO compounds immediately prior to reperfusion. There was a considerable period of time between the earliest study in 1992 and the most recent study in 2014. Measurement of infarct size in each of the studies was performed in a different way. Enzyme release into plasma was used in the earlier studies to measure CK-MB or HBDH [21, 37] whilst cardiac magnetic ressonance (CMR) was used in the 2014 NIAMI trial [59]. Unlike the experimental setting where infarct size measurement is reliably measured by post mortem histological staining and direct imaging techniques, there is as yet no consistent, gold standard technique for assessing infarct size relative to risk zone size in the clinical setting [20].

A reperfusion protocol formed part of the inclusion criteria in this review. However both Hildebrandt et al. [21] and Morris et al. [37] performed subgroup analysis on patients in which thrombolysis was ineffective or reperfusion was limited. Hildebrandt et al. [21] reported in this sub group of patients that isosorbide dinitrate did afford some infarct limitation. Morris et al. [37] however, suggest that in their sub-group analysis of patients with incomplete reperfusion, judged by ST segment resolution, isosorbide dinitrate had no effect on infarct size. They further reported that patients with an intermediate ST elevation benefited from isosorbide dinitrate in contrast to patients with large ST elevation in which isosorbide dinitrate was deleterious. Siddiqi et al. [59] reported that infarct size in their patients was relatively large compared to placebo treated patients in a remote conditioning study from 2010, yet there was no relationship between patients with smaller or larger infarcts, varying risk areas or chest pain duration.

All clinical studies were conducted double-blind. In all studies patient populations were heterogeneous, with similar mean ages and sex distribution. In each of these studies, infarct size, time to reperfusion, age, and the presence of comorbidities was variable. This is a criticism of translational science generally, which may in part explain the disparity between clinical and animal studies. The animal studies included in this review reported data from healthy juvenile animals with no comorbidities and highly regulated infarct size and location. The clinical trials reviewed here, like most others, have a study cohort of patients with numerous comorbidities and, as the current clinical studies report, varying degrees of infarct size, location and indeed reperfusion success. A recent phase 2 trial which was published after our literature screening and analysis, in which 82 patients were randomized to sodium nitrite or placebo just prior to PPCI reflected the outcomes of the human trials included in this review. No reduction in infarct size was observed, measured by CK and troponin and subsequent CMR. However a reduction in major adverse cardiac events was reported [28].

The complexity and number of comorbidities that present alongside AMI create significant challenges when translating therapies to the clinic. Disruption to cytoprotecitve signalling as a consequence of metabolic disturbances and other pathological processes have commanded much interest due to unsuccessful translation of cardioprotection strategies. Indeed, of particular interest to NOx signalling is the downstream sGC associated kinase PKG. The protection afforded by NO donor SNAP was abolished in a hyperlipidaemic rat model, via possible oxidative dimerization of PKG in rats fed a cholesterol rich diet [11]. Similarly, diabetes has been shown to impair pharmacological postconditioning in an in vivo rabbit model. Isoflurane induced infarct size limitation was abrogated in hyperglycaemic rabbits, which was associated with impaired Akt/eNOS signalling [51].

Concomitant pharmacotherapy with pharmacological agents such as antihypertensives, anti-anginal drugs, lipid-lowering drugs, anti-platelet aspirin, and drugs used for the treatment of diabetes among others, modify the signalling cascades that are of interest to limit the injurious effects of AMI and may also confound clinical studies. For example, statins have been extensively studied in both animal models and in humans. Although there is a large body of evidence that suggests that many statins positively modify NO signalling via eNOS induction (comprehensively reviewed in [48]), pravastatin demonstrated opposing effects on myocardial NO levels [24]. Many of these therapies may provide protection against irreversible injury and so additional intervention will only induce small incremental limitation of infarct size [9]. At high micromolar and millimolar concentrations NO can promote cellular injury, a situation that is possible in patients being treated with polypharmacy. Therefore it is essential to define the optimum compound, formulation and dose to minimise toxicity of these compounds when administered in clinical AMI. Timing and administration route are also crucial considerations which may be possible when mechanisms are further understood.

## Conclusion

All NO donor agents except NTG exhibit the potential to limit infarct size when given as adjuncts to reperfusion in various in vivo animal models of ischemia/reperfusion. Despite this there is no definitive conclusion to the exact mechanism(s) by which beneficial effects are obtained. The evidence reported in this review emphasises a disparity between preclinical animal studies and the human trials. It is clear that the preclinical models included for review here, do not reflect the complexities and heterogeneity of the human cohort. The lack of standardised infarct size measurement relative to risk zone, marked variation in time to reperfusion/intervention and variation in the ischemic territory all present challenges to assessment of adjunct therapies. Further well designed pre-clinical models which better reflect the complexities of the human setting and subsequent high quality RCTs are needed.
